# Practice variation in the treatment of patients with renal hyperparathyroidism: a survey-based study in the Netherlands

**DOI:** 10.1186/s12882-021-02361-7

**Published:** 2021-04-23

**Authors:** Jaimie L. H. Zhang, Natasha M. Appelman-Dijkstra, Edouard L. Fu, Joris I. Rotmans, Abbey Schepers

**Affiliations:** 1 Department of Surgery, Leiden University Medical Center, Leiden, Netherlands; 2 Department of Internal Medicine division Endocrinology, Leiden University Medical Center, Leiden, Netherlands; 3 Department of Clinical Epidemiology, Leiden University Medical Center, Leiden, Netherlands; 4 Department of Internal Medicine division Nephrology, Leiden University Medical Center, Leiden, Netherlands

**Keywords:** Hyperparathyroidism, Chronic kidney failure, Chronic kidney metabolic bone disorder, Parathyroidectomy, Cinacalcet

## Abstract

**Background:**

Renal hyperparathyroidism is a disease entity that is complex and poorly understood. Although there are guidelines regarding how to manage this patient group, evidence is scarce. Therefore, this survey-based study aims to map the physicians’ attitude in terms of preference for management of renal hyperparathyroidism and the influence of patient and respondent factors.

**Methods:**

A survey was sent to Dutch societies of nephrology, endocrinology, and surgeons with interest in endocrine surgery. The survey consisted of eight case vignettes of renal hyperparathyroidism patients who were on hemodialysis and suitable for kidney transplantation, and varied in one of three patient variables import for decision making: age (40 vs. 65 years), parathyroid hormone (40 vs. 90 pmol/L), and serum calcium level (2.25 vs. 2.8 mmol/L). For each case, respondents could choose between maintaining conservative treatment (active vitamin D metabolites), calcimimetics, or subtotal parathyroidectomy as their treatment of choice. Categorical multilevel logistic models were used to investigate the association of patient and respondent variables with treatment preference. The influence of patient variables was determined independently of each other and by means of logistic regression the probabilities of treatment choice were calculated.

**Results:**

In total, 115 surveys were included in the analysis. In 6 out of 8 cases, less than two-thirds of respondents agreed on the most favoured treatment. Among patient characteristics, the main disincentive for respondents not to choose conservative therapy was an elevated serum calcium level (subtotal parathyroidectomy vs conservative OR 93.1, 95%-CI: 48.39–179.07 and calcimimetics vs conservative OR 31.2 95%-CI: 18.58–52.30). Additionally, the most significant treatment differences were found between medical specialties and the experience of the respondents, expressed as the amount of cases the physician was involved in during the past year.

**Conclusions:**

Elevated serum calcium levels were widely recognized and the prime reason for respondents to abandon conservative treatment. However, considerable disagreement in treatment preferences remained throughout the cases, demonstrating the current literature available being inconclusive in guiding physicians. Therefore, a high-quality trial comparing subtotal parathyroidectomy to medical treatment is needed to determine optimal treatment.

**Supplementary Information:**

The online version contains supplementary material available at 10.1186/s12882-021-02361-7.

## Introduction

One of the most common and early metabolic disorders in chronic kidney disease (CKD) is renal hyperparathyroidism (RHPT). RHPT can be divided into secondary hyperparathyroidism (SHPT) and tertiary hyperparathyroidism (THPT). The former is usually diagnosed at first in patients with CKD and is characterized by elevated parathyroid hormone (PTH) levels as a result of the derangements in the homeostasis of phosphate, vitamin D, and, in later stages, also calcium. Approximately 30–50% of end-stage kidney disease patients have SHPT [1]. During the course of chronic renal failure, SHPT can develop into THPT in which, presumably because of prolonged stimulation, the parathyroid gland proliferates, autonomously secretes PTH regardless of feedback and thereby leading to hypercalcemia [2, 3]. RHPT pathophysiology is complex as many pathogenetic peculiarities are not defined nor understood. This emerges from the dilemma wherein RHPT-patients refractory to conservative therapy, meaning active vitamin D metabolites, require high doses of activated vitamin D despite the potential to cause hypercalcemia in SHP. Methods within the treatment arsenal of a physician in disrupting this vicious cycle are either parathyroidectomy (PTx) or calcimimetics [4]. Calcimimetics are considered an acceptable bridge to renal transplantation or treatment in non-operable patients because it is effective even in patients with marked parathyroid hyperplasia [5]. Besides decreasing PTH and calcium levels, cinacalcet has also been shown to be effective in improving bone-turnover and histology, [6] and phosphate levels including patients on haemodialysis [7]. However, the effects of calcimimetics on fracture rate, major cardiovascular events or mortality remain controversial. Regarding PTx, evidence is lacking on its superiority over medical treatment since only observational studies have been carried out thus far. Moreover, the disparity between increasing PTH levels in dialysis patients with simultaneous decrease of PTx performed pleads for a prevailing uncertainty about optimal PTH targets [8, 9].

Despite the advances in the therapeutic armamentarium available for the management of RHPT, clear guidelines of therapeutic targets and primary outcomes are still lacking [10]. To date, no studies have been published mapping the treatment-related attitude of the physicians involved in managing RHPT patients. This study therefore aimed to investigate the physicians’ preference for either a conservative, calcimimetics, or surgical policy for the management of RHPT in the Netherlands. Additionally, we aimed to identify the factors influencing these preferences and to assess differences in treatment preferences across specialties and other physician subgroups. We hypothesized that a high level of variation in treatment preference will be present due to the lack of an above-mentioned guideline.

## Methods

A study group consisting of an endocrinologist, nephrologist, and endocrine surgeon (authors: NMA-D, JIR, and AS) at the Leiden University Medical Center reached consensus regarding the most crucial factors influencing physicians’ decisions concerning renal hyperparathyroidism-related patients. Using these factors, the questionnaire was drafted and sent to all physicians associated with the Dutch Society of Nephrology, the Dutch Society of Endocrinology and the members of the Dutch Hyperparathyroid Study Group, who were asked to participate in this study. In the questionnaire respondents were requested to state their professional characteristics including medical specialty, number of RHPT related treatment decisions per year, seniority, and affiliation. Thereafter, eight case vignettes were presented of hypothetical patients, being screened for kidney transplantation and maintained under optimized 25 (OH) vitamin D and plasma phosphate levels (Table 1). Case vignettes were presented with all possible combinations of age, serum parathyroid hormone (PTH) and calcium. For each case, respondents were asked to state whether they preferred maintaining conservative treatment (active vitamin D metabolites), treatment with calcimimetics or subtotal parathyroidectomy.

**Table 1:** Example case vignette. Age, serum calcium, and PTH were varied for the eight clinical case vignettes

40-year old male		
Currently on hemodialysis for 1.5 years, being screened for kidney transplantation in order to receive a donor kidney from his brother. Serum PTH and calcium: **40 pmol/L** (ref: 0.7–8 pmol/L) and **2.25 mmol/L** (ref: 2.15–2.55 mmol/L) respectively. 25 (OH) Vitamin D and plasma phosphate are 100 nmol/L (ref: > 50 nmol/L) and 1.4 mmol/L (ref: 0.9–1.5 pmol/L) respectively, optimized by vitamin D supplementation and phosphate binders.		
What would be your treatment of choice for this patient?		
Maintain conservative treatment	Start calcimimetic treatment	Opt for subtotal parathyroidectomy

Case vignettes were presented in a chronological order from case 1 to 8. A response to each multiple-choice question was required to continue to the next case. Upon completion of the 8 case vignettes, respondents were presented with one final case vignette of the RHPT patient presented in Table 1, but without PTH concentration. Respondents were asked above which PTH concentration they would opt for a subtotal PTx. As a final question, respondents could comment about whether they would have considered other variables of importance than the ones stated and why.

The invitation to participate in the online questionnaire was sent out as a separate e-mail or in the societies’ newsletters. A reminder was sent 4 weeks after the first call. The survey was drafted in SurveyMonkey® and can be found in the Supplementary Materials.

### Statistical analysis

Descriptive statistics were used to describe the respondents’ characteristics. Respondents who did not answer any case vignette were excluded from the analysis. Partially filled-in case vignettes were included.

We assessed the association between case- or respondent related factors and the respondents’ treatment preferences (conservative treatment, vs. calcimimetics vs. subtotal PTx) using categorical multilevel logistic models [11]. Categorical multilevel logistic models can be used to estimate the respondents’ preference of choosing either treatment option in comparison to another while accounting for the fact that a respondents’ answers across the cases are correlated. The multilevel logistic model was used to estimate odds ratios (OR), 95% confidence intervals and to calculate probabilities of choosing either treatment as the result of each patient or respondent-related variable. The influence of a patient/case variable on treatment preference was calculated independent of the remaining 2 case variables. The case variables included patient age (40 vs. 65 years), calcium levels (2.25 vs. 2.8 mmol/L) and PTH-level (40 vs. 90 pmol/L). Respondent-related factors included medical specialty (nephrology, surgery, endocrinology), respondent’s experience and affiliation (academic vs affiliated). Respondents’ experience was expressed in both the number of RHPT-related treatment decisions made in the past year and years of practicing the current specialty. Both were dichotomized in a group below the median number, and the median number and above group.

Analyses were performed in SPSS version 25 (SPSS, IBM Corporation).

## Results

### Participants

The total number of physicians participating was 136, of whom 21 did not provide answer to any of the case vignettes and were therefore excluded. Characteristics of included and excluded respondents are reported in Table 2. The 115 included respondents consisted of 73 (63.5%) nephrologists, 25 (21.7%) surgeons and 17 (14.8%) endocrinologists. They had a median of 10 years (IQR: 5–20 years) of clinical experience and were involved in a median of 20 RHPT-related treatment decisions in the past year (IQR: 5–50 cases). Forty-two (36.5%) respondents were affiliated with an academic hospital. Seven respondents (6.1%) stated to not have been involved in any RHPT-related treatment decision during the past year. Of the excluded respondents, there seemed to be a proportionally high number of endocrinologists (10 of 21) and the involvement in RHPT-related treatment decisions in the past year was lower (median of 5 cases).

**Table 2:** Respondent characteristics

		Included (*n* = 115)	Excluded (*n* = 21)
Specialty	Nephrology	73 (63.5%)	4 (19.0%)
	Surgery	25 (21.7%)	6 (28.6%)
	Endocrinology	17 (14.8%)	10 (47.6%))
	Other	–	1 (4.8%)
Years of experience		10 (5; 20)	10 (3.5; 19)
Renal HPT-related decisions in the past year		20 (5; 50)	5 (5; 17.5)
Affiliation	Afiliated hospital	73 (63.5%)	11 (52.4%)
	Academic hospital	42 (36.5%)	10 (47.6%)

### Treatment preferences

**Fig. 1:**
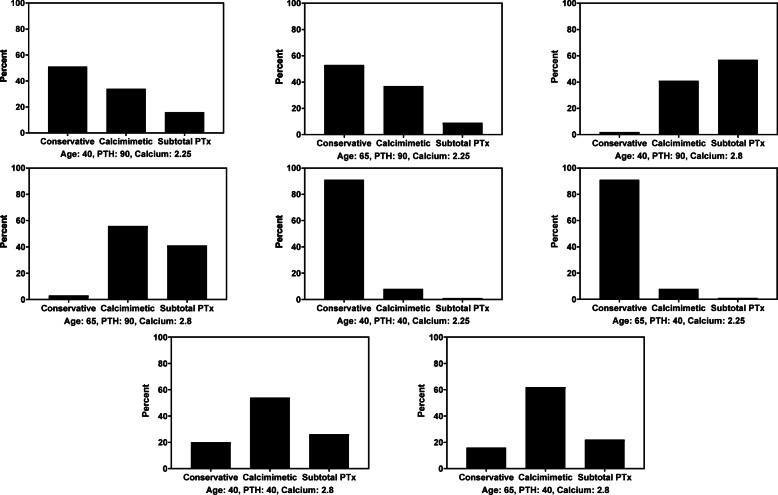
Probabilities of treatment choice based on logistic regression of the independently calculated patient/case variables (age in years, PTH in pmol/L, and calcium in mmol/L)

The cases characterized by a mildly elevated PTH (40 pmol/L) and normocalcemia (2.25 mmol/L) had an evidently higher consensus: for both ages there was a 91% calculated probability of respondents choosing conservative treatment (Fig. 1). Although close with other cases, the most variability was evident in cases characterized by high PTH (90 pmol/L) and normocalcemia (2.25 mmol/L): the consensus was 51 and 53% for respectively the younger and older patient. Similar to the previous cases with the highest consensus, conservative treatment was the most favored policy.

### Impact of patient characteristics on treatment choice

Increasing age independently from PTH and calcium resulted in a significant decrease of respondents opting for subtotal PTx instead of conservative treatment (OR 0.55, 95%-CI: 0.33–0.93), while age did not seem to have a significant impact when comparing calcimimetics to conservative treatment: (OR 1.04, 95%-CI: 0.69–1.58). Higher PTH and serum calcium levels were both independently associated with treatment choice (subtotal PTx vs conservative OR 21.6, 95%-CI: 11.74–39.66; OR 93.1, 95%-CI: 48.39–179.07; calcimimetics vs conservative OR 7.54, 95%-CI: 4.66–12.20; OR 31.2 95%-CI: 18.58–52.30, respectively, Table 3). In both treatment-comparing considerations, an increase in calcium was the driver for refraining from choosing conservative treatment.

**Table 3:** Odds ratios for the association between case and respondent variables and treatment preference using a multilevel categorical logistic model. Subtotal PTx and calcimimetics were compared to conservative treatment (reference category)

Predictor	OR	95%-CI	*p*-value
Patient/Case variables			
Age: 65 years^a^			
Subtotal PTx	0.55	0.33–0.93	**0.02**
Calcimimetics	1.04	0.69–1.58	0.84
PTH: 90 pmol/L^b^			
Subtotal PTx	21.6	11.74–39.66	**< 0.001**
Calcimimetics	7.54	4.66–12.20	**< 0.001**
Calcium: 2.8 mmol/L^c^			
Subtotal PTx	93.1	48.39–179.07	**< 0.001**
Calcimimetics	31.2	18.58–52.30	**< 0.001**
Respondent variables			
Surgeon^d^			
Subtotal PTx	1.44	0.69–3.01	0.33
Calcimimetics	0.55	0.34–0.90	**0.02**
Endocrinologist^e^			
Subtotal PTx	2.56	1.14–5.72	**0.02**
Calcimimetics	0.54	0.29–0.96	**0.04**
At and above RHPT-related decisions^f^			
Subtotal PTx	0.45	0.24–0.81	**0.009**
Calcimimetics	1.29	0.87–1.91	0.21
At and above years of experience^g^			
Subtotal PTx	1.11	0.60–2.05	0.73
Calcimimetics	1.3	0.88–1.93	0.19
Affiliated^h^			
Subtotal PTx	1.15	0.61–2.16	0.66
Calcimimetics	1.19	0.79–1.78	0.41

(a) reference category: age 40 years, (b) reference category: PTH 40 pmol/L, (c) reference category: calcium 2.25 mmol/L, (d) reference category: nephrologists, (e) reference category: nephrologists, (f) reference category: below the median of RHPT-related treatment decisions in the past year, (g) reference category: below the median years of experience in the current specialty, and (h) reference category: academic hospital

### Impact of respondent characteristics on treatment choice

**Fig. 2:**
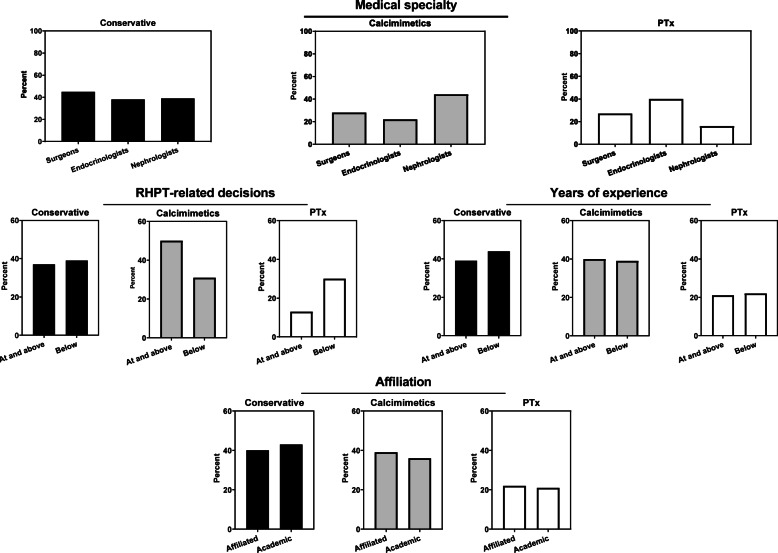
Probabilities of treatment choice based on logistic regression of the respondent variables

Firstly, the association between affiliation and treatment preference was investigated from which emerged that the odds was 2.56 times higher for endocrinologists as supposed to nephrologists in opting for subtotal PTx instead of conservative treatment (OR 2.56, 95%-CI: 1.14–5.72, Table 3). No statistically significant difference was found when comparing surgeons with nephrologists in the same regard (OR 1.44, 95%-CI: 0.69–3.01). Compared with nephrologists, both surgeons and endocrinologists were less likely to choose calcimimetics than conservative treatment (OR 0.55, 95%-CI: 0.34–0.90; OR 0.54 95%-CI: 0.23–1.33, respectively). Therefore, as a specialty, nephrologists seemed to have a relatively high tendency to opt for calcimimetics instead of conservative treatment. This is reflected in Fig. 2, where probabilities of choosing either treatment policy were categorized by medical specialism: nephrologists were calculated to have a relatively high probability to opt for calcimimetics, mainly at the expense of choosing subtotal PTx. On the other hand, the aggressiveness in treatment of RHPT-related patients of endocrinologists became apparent from the calculated probability of 40% for subtotal PTx.

Next, analyses were performed based on the respondents’ experience. The dichotomization of the respondents by the number of RHPT-related treatment decisions exhibited the at or above the median group to be significantly less inclined to choose for subtotal PTx instead of conservative treatment than the below the median group (OR 0.45, 95%-CI: 0.24–0.81, Table 3). In contrary, when comparing the two groups regarding calcimimetics versus conservative treatment, high volume respondents were found to relatively frequently treat with calcimimetics (OR 1.29, 95%-CI: 0.87–1.91). These findings were reflected in the probabilities: the high-volume group had a relatively high probability of opting for calcimimetics at the expense of subtotal PTx (calcimimetics: 50% vs 31%, subtotal PTx 13% vs 30%, respectively, Fig. 2). The number of years the respondent practiced the current specialty was an alternative measure for experience but did not show any significant nor strong effects expressed in OR. Therefore, the frequency of RHPT-related treatment decisions apparently led to more differentiation in the respondents’ attitude than years practicing the current specialty as seen in the probabilities. Finally, an analysis was performed between affiliated and academic-related respondents. No significant influence of affiliation on treatment preferences was observed.

In the original results as well as throughout all sub-analyses, the case containing age: 65, mildly elevated PTH (40 pmol/L) with normocalcemia (2.25 mmol/L) had the highest consensus.

### Influence of PTH-concentration on the tendency to PTx

**Fig. 3:**
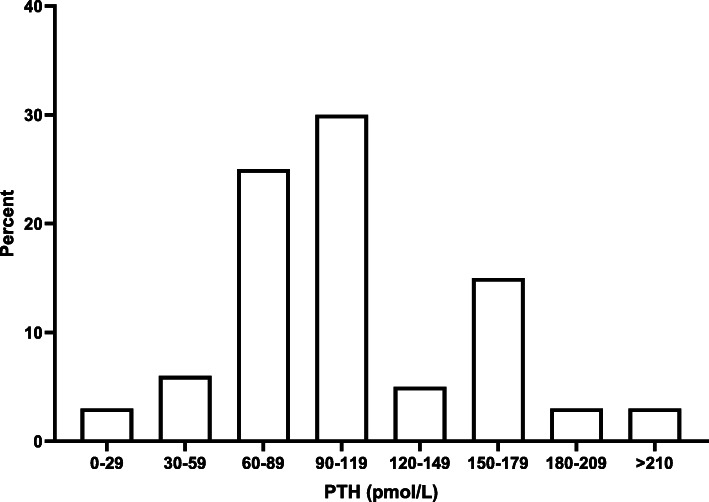
Cut-off value of PTH concentration (pmol/L) above which subtotal PTx is preferred by respondents for a case with variables age: 40 years and calcium: 2.25 mmol/L

**Fig. 4:**
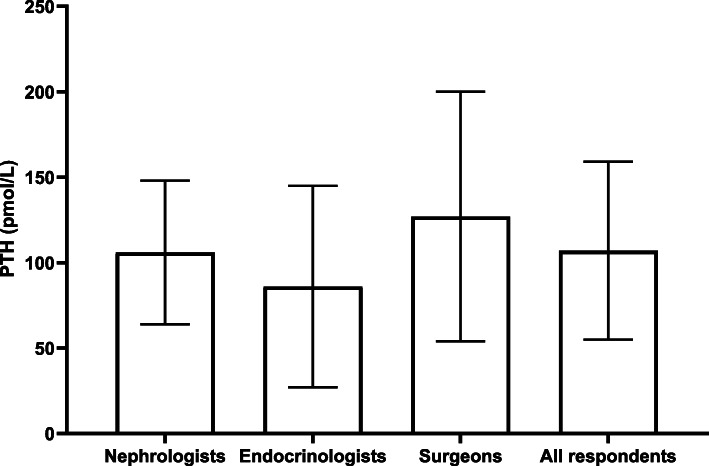
PTH concentration (pmol/L ± standard deviation) above which nephrologists (*n* = 59), endocrinologists (*n* = 14) and surgeons (*n* = 17) would opt for a PTx

The mean PTH concentration (pmol/L) in which respondents chose to opt for a subtotal PTx in a 40-year old patient with a serum calcium of 2.25 mmol/L was 107 ± 52 pmol/L (Fig. 3). Based on the mean cut-off value in the sub-analysis of this question, surgeons (*n* = 17) have the lowest tendency to opt for subtotal PTx with a high variance (125 ± 73 pmol/L). On the contrary, endocrinologists (*n* = 14) seemed to have the highest tendency (106 ± 4 pmol/L) (Fig. 4).

### Other relevant factors for decision making

Respondents could comment on which factors, other than the ones already stated in the questionnaire, they deemed important in their decision-making process. The most frequently encountered answer was responsiveness to conservative/calcimimetics treatment (*n* = 16), results of screening for other causes of hypercalcemia (*n* = 6), PTH stability (*n* = 5), eligibility of patient for kidney donation (*n* = 5) and the presence of complications due to hypercalcemia (*n* = 5).

## Discussion

This study aimed to investigate the physicians’ treatment preference for the management of RHPT and the influence of patient and respondent factors on treatment preferences. As hypothesized, there was lacking consensus on how to optimally treat patients suffering from RHPT. Our study shows that as biochemical risk factors of cases increased, disparity regarding treatment became progressive.

The results reflect the current state of literature wherein the impact of PTH versus serum calcium levels in defining operative indications is poorly described. Especially patients in whom not necessarily both values of calcium and PTH levels are elevated, there is dissent regarding which value is more indicative of a need for subtotal PTx. Opinions are even divided regarding whether to include elevated serum PTH in the analysis, as an elevation of exclusively PTH does not exhibit obvious symptoms as hypercalcemia often does and likewise normalization of PTH is not generally viewed as an essential metric of success in the same way that normocalcemia is. Subtotal PTx has been associated with improved survival in observational studies of large dialysis cohorts, with reported reduction in all-cause mortality of 15–57% [12–16]. For this reason, subtotal PTx seems indicated/appropriate in the presence of persistent hypercalcemia or -phosphatemia even without a defined PTH threshold and even more so when the patient is refractory to medical therapy. Although promising, these studies were observational and thus subject to confounding by indication, heterogeneity of patients, differences in types of dialysis, etc. Chances for a trial comparing subtotal PTx to medical treatment are viewed to be low due to potential surgical morbidity, hospitalization costs, and increasing age and comorbidities among patients on dialysis. However, the threshold of conducting such a study should not be high due to the expected long-term cost-effectiveness of subtotal PTx over calcimimetics [17]. The European Society of Endocrine Surgeons stated in a consensus report of 2015, that subtotal PTx was specifically indicated when medical treatment fails to correct metabolic parameters meaning hypercalcemia, hyperphosphatemia, but interestingly also PTH > 85 pmol/L. [18].

Among the 3 patient variables used, our results imply physicians finding calcium to be the main driver in this consideration. Nevertheless, calculated probabilities showed an independent rise in PTH also leading to a shift in treatment preference from conservative to mainly calcimimetics in numerical manner, but also to subtotal PTx. Moreover, the question wherein physicians could choose a PTH concentration where they would opt for a subtotal PTx in a 40-year old patient with a serum calcium of 2.25 mmol/L also shows that the PTH level of a patient should not be neglected. A low OR was observed for choosing subtotal PTx instead of conservative treatment when the patient age rose from 40 to 65 years. Clinically, age seemed to matter only in a physician’s preference of dealing with RHPT when the biochemical values were progressive.

Nephrologists, as compared to endocrinologists and endocrine surgeons, seemed to have a high tendency to refrain from treating patients with subtotal PTx and start treatment with calcimimetics instead. The opposite was seen among endocrinologists. This could be explained by endocrinologists and endocrines surgeons being accustomed to treating patients with hyperparathyroidism, meaning not just those with a renal cause. Across their whole patient group, PTx occurs more frequently than just for those suffering from RHPT. An alternative explanation could be the current perspective of nephrologists coming about as a result of their dealing with patients more abundant in mortality/risks for surgery.

Furthermore, a positive correlation was found between the amount of RHPT-related treatment decisions per year and refraining from choosing subtotal PTx. This could be explained by the natural drive of less experienced clinicians to act upon mainly progressive biochemical values. Since deciding which treatment is correct is out of the scope of this study, it remains unclear whether the phenomenon of refrainment in treating with subtotal PTx in more experienced physicians is justified.

Since consensus regarding the best treatment for RHPT is lacking, shared decision making is of the utmost importance. In order to properly counsel patients on this complex topic, physicians should have a thorough understanding of the pathophysiology of RHPT even in the light of awaiting a kidney transplant and the pros of cons of each treatment modality.

### Limitations

Important limitations of this study are the simplification of the cases, necessary to obtain robust and indicative results and the likelihood of the answers of individual respondents not directly reflecting the treatment decisions made in clinical practice. In this complex patient group, treatment choices would be made in a multidisciplinary context, taking into account even more variables. Furthermore, this study reflects the disagreement among physicians in the Netherlands, and it remains unclear whether these results can be extrapolated to other Western countries.

## Conclusion and future perspective

Despite a rise in serum calcium being the main driver for abandoning conservative treatment in the management of RHPT patients and, thereafter, increase in PTH level, considerable disagreement remained and demonstrates the current evidence available not being convincing. From the analysis of respondent variable influence on decision making, it appeared that there was significant difference in treatment preference amongst medical specialties and experience expressed in amount of RHPT-related cases treated in the past year. Therefore, we hope this research will stimulate discussion and shed a light on the considerations and optimal care for RHPT patients. In the hope for more consensus among physicians in dealing with this difficult patient-group we, despite all disincentives, strongly plead for a trial comparing subtotal PTx to medical treatment as it would be definite in the question whether either is better in what situation.

## Supplementary Information

**Additional file 1.**

**Additional file 2.**

## Data Availability

The datasets used and/or analysed during the current study are available from the corresponding author on reasonable request.

## Abbreviations

CKD: Chronic kidney disease
OR: Odds-ratio
PTH: Parathyroid hormone
PTx: Subtotal parathyroidectomy
RHPT: Renal hyperparathyroidism
SHPT: Secondary hyperparathyroidism
THPT: Tertiary hyperparathyroidism
