# Supplementary material for: Practice variation in the treatment of patients with renal hyperparathyroidism: a survey-based study in the Netherlands
Source: BMC Nephrol. 2021 Apr 23;22:150. doi: 10.1186/s12882-021-02361-7 (PMC8066971; doi:10.1186/s12882-021-02361-7)
Supplement: Supplementary file 1 — Additional file 1. [file 12882_2021_2361_MOESM1_ESM.pdf]

Dutch Hyperparathyroidism / Hypoparathyroidism Study Group

Survey on the treatment of hyperparathyroidism in patients with ESKD

Introduction

Dear colleague,

You have reached our survey investigating the care for dialysis patients with hyperparathyroidism. This survey will take approximately 6 minutes to complete. With your answers, we aim to investigate the current clinical practice and factors influencing hyperparathyroidism-related decisions in this group of patients.

You will be presented 8 unique short clinical cases concerning hyperparathyroidism in dialysis patients suitable for transplantation. All cases are the same, except for 3 aspects:

- **Age**
- **PTH level**
- **Type of hyperparathyroidism**

After each case you will be asked whether you would opt for the following in the decision-making process:

- **Maintain conservative treatment**
- **Treatment with calcimimetics**
- **Subtotal parathyroidectomy**

Optionally, you may provide comments for each case.
We would like to thank you in advance for your participation in this survey.

Yours sincerely,

On behalf of the Dutch Hyperparathyroid Study Group,
Abbey Schepers (endocrine surgeon)
Natasha Appelman (endocrinologist)
Joris Rotmans (nephrologist)
Jaimie Zhang (researcher)

* 1. What is your speciality?

- Nephrology (in training)
- Endocrinology (in training)
- Surgery (in training)
- Other (please specify)

* 2. For how many years have you been practicing your current specialty?

* 3. How many renal hyperparathyroidism-related treatment decisions were you involved with during the past year?

* 4. What is your affiliation?

- Acedemic hospital
- Affiliated hospital
- Other (please specify)

* 5. In which country?

Dutch Hyper/Hypo parathyroidism Study Group

Survey on the treatment of hyperparathyroidism in patients with ESKD

Case 1

40-year old male
Currently on hemodialysis for 1.5 years, being screened for kidney transplantation in order to receive a donor kidney from his brother.
Serum PTH and calcium: 90 pmol/L (ref: 0.7-8 pmol/L) and 2.25 mmol/L (ref: 2.15-2.55 mmol/L) respectively.
25 (OH) Vitamin D and plasma phosphate are 100 nmol/L (ref: > 50 nmol/L) and 1.4 mmol/L (ref: 0.9-1.5 pmol/L) respectively, optimized by vitamin D supplementation and phosphate binders.

* 1. What would be your treatment of choice for this patient?

Maintain conservative treatment	Start calcimimetic treatment	Opt for subtotal parathyroidectomy
○	○	○

2. Comment (optional)

Dutch Hyper/Hypo parathyroidism Study Group

Survey on the treatment of hyperparathyroidism in patients with ESKD

Case 2

65-year old male
Currently on hemodialysis for 1.5 years, being screened for kidney transplantation in order to receive a donor kidney from his brother.
Serum PTH and calcium: 90 pmol/L (ref: 0.7-8 pmol/L) and 2.25 mmol/L (ref: 2.15-2.55 mmol/L) respectively.
25 (OH) Vitamin D and plasma phosphate are 100 nmol/L (ref: > 50 nmol/L) and 1.4 mmol/L (ref: 0.9-1.5 pmol/L) respectively, optimized by vitamin D supplementation and phosphate binders.

* 1. What would be your treatment of choice for this patient?

Maintain conservative treatment	Start calcimimetic treatment	Opt for subtotal parathyroidectomy
○	○	○

2. Comment (optional)

Survey on the treatment of hyperparathyroidism in patients with ESKD

Case 3

40-year old male
Currently on hemodialysis for 1.5 years, being screened for kidney transplantation in order to receive a donor kidney from his brother.
Serum PTH and calcium: 90 pmol/L (ref: 0.7-8 pmol/L) and 2.8 mmol/L (ref: 2.15-2.55 mmol/L) respectively.
25 (OH) Vitamin D and plasma phosphate are 100 nmol/L (ref: > 50 nmol/L) and 1.4 mmol/L (ref: 0.9-1.5 pmol/L) respectively, optimized by vitamin D supplementation and phosphate binders.

* 1. What would be your treatment of choice for this patient?

Maintain conservative treatment	Start calcimimetic treatment	Opt for subtotal parathyroidectomy
○	○	○

2. Comment (optional)

Dutch Hyper/Hypo parathyroidism Study Group

Survey on the treatment of hyperparathyroidism in patients with ESKD

Case 4

65-year old male
Currently on hemodialysis for 1.5 years, being screened for kidney transplantation in order to receive a donor kidney from his brother.
Serum PTH and calcium: 90 pmol/L (ref: 0.7-8 pmol/L) and 2.8 mmol/L (ref: 2.15-2.55 mmol/L) respectively.
25 (OH) Vitamin D and plasma phosphate are 100 nmol/L (ref: > 50 nmol/L) and 1.4 mmol/L (ref: 0.9-1.5 pmol/L) respectively, optimized by vitamin D supplementation and phosphate binders.

* 1. What would be your treatment of choice for this patient?

Maintain conservative treatment	Start calcimimetic treatment	Opt for subtotal parathyroidectomy
○	○	○

2. Comment (optional)

Dutch Hyper/Hypo parathyroidism Study Group

Survey on the treatment of hyperparathyroidism in patients with ESKD

Case 5

40-year old male
Currently on hemodialysis for 1.5 years, being screened for kidney transplantation in order to receive a donor kidney from his brother.
Serum PTH and calcium: 40 pmol/L (ref: 0.7-8 pmol/L)and 2.25 mmol/L (ref: 2.15-2.55 mmol/L) respectively.
25 (OH) Vitamin D and plasma phosphate are 100 nmol/L (ref: > 50 nmol/L) and 1.4 mmol/L (ref: 0.9-1.5 pmol/L) respectively, optimized by vitamin D supplementation and phosphate binders.

* 1. What would be your treatment of choice for this patient?

Maintain conservative treatment	Start calcimimetic treatment	Opt for subtotal parathyroidectomy
○	○	○

2. Comment (optional)

Dutch Hyper/Hypo parathyroidism Study Group

Survey on the treatment of hyperparathyroidism in patients with ESKD

Case 6

65-year old male

Currently on hemodialysis for 1.5 years, being screened for kidney transplantation in order to receive a donor kidney from his brother.

Serum PTH and calcium: 40 pmol/L (ref: 0.7-8 pmol/L) and 2.25 mmol/L (ref: 2.15-2.55 mmol/L) respectively.

25 (OH) Vitamin D and plasma phosphate are 100 nmol/L (ref: > 50 nmol/L) and 1.4 mmol/L (ref: 0.9-1.5 pmol/L) respectively, optimized by vitamin D supplementation and phosphate binders.

* 1. What would be your treatment of choice for this patient?

Maintain conservative treatment	Start calcimimetic treatment	Opt for subtotal parathyroidectomy
◯	◯	◯

2. Comment (optional)

Dutch Hyper/Hypo parathyroidism Study Group

Survey on the treatment of hyperparathyroidism in patients with ESKD

Case 7

40-year old male
Currently on hemodialysis for 1.5 years, being screened for kidney transplantation in order to receive a donor kidney from his brother.
Serum PTH and calcium: 40 pmol/L (ref: 0.7-8 pmol/L) and 2.8 mmol/L (ref: 2.15-2.55 mmol/L) respectively.
25 (OH) Vitamin D and plasma phosphate are 100 nmol/L (ref: > 50 nmol/L) and 1.4 mmol/L (ref: 0.9-1.5 pmol/L) respectively, optimized by vitamin D supplementation and phosphate binders.

* 1. What would be your treatment of choice for this patient?

Maintain conservative treatment	Start calcimimetic treatment	Opt for subtotal parathyroidectomy
○	○	○

2. Comment (optional)

Survey on the treatment of hyperparathyroidism in patients with ESKD

Case 8

65-year old male
Currently on hemodialysis for 1.5 years, being screened for kidney transplantation in order to receive a donor kidney from his brother.
Serum PTH and calcium: 40 pmol/L (ref: 0.7-8 pmol/L) and 2.8 mmol/L (ref: 2.15-2.55 mmol/L) respectively.
25 (OH) Vitamin D and plasma phosphate are 100 nmol/L (ref: > 50 nmol/L) and 1.4 mmol/L (ref: 0.9-1.5 pmol/L) respectively, optimized by vitamin D supplementation and phosphate binders.

* 1. What would be your treatment of choice for this patient?

Maintain conservative treatment	Start calcimimetic treatment	Opt for subtotal parathyroidectomy
○	○	○

2. Comment (optional)

Dutch Hyper/Hypo parathyroidism Study Group

Survey on the treatment of hyperparathyroidism in patients with ESKD

40-year old male
Currently on hemodialysis for 1.5 years, being screened for kidney transplantation in order to receive a donor kidney from his brother.
Hyperparathyroidism and calcium 2.25 mmol/L (ref: 2.15-2.55 mmol/L).
25 (OH) Vitamin D and plasma phosphate are 100 nmol/L (ref: > 50 nmol/L) and 1.4 mmol/L (ref: 0.9-1.5 pmol/L) respectively, optimized by vitamin D supplementation and phosphate binders.

* 1. Above which PTH concentration would you opt for a subtotal parathyroidectomy?

0 pmol/L 300

Dutch Hyper/Hypo parathyroidism Study Group

Survey on the treatment of hyperparathyroidism in patients with ESKD

Optional question

In any of the previous cases, would you have considered other variables of importance than the ones mentioned?

- **If you would have considered other variables, please explain why.**

1. Comments

Dutch Hyper/Hypo parathyroidism Study Group

Survey on the treatment of hyperparathyroidism in patients with ESKD

Thank You

Thank you for your participation in our survey.
To submit your answers, please click the "Finish" button below.

Optionally, you may leave comments on the survey in the comment box below.
Alternatively, if you would like to receive the survey results, please leave your e-mail address.

1. Comments/E-mail
