# Supplementary material for: Practice variation in the treatment of patients with renal hyperparathyroidism: a survey-based study in the Netherlands
Source: BMC Nephrol. 2021 Apr 23;22:150. doi: 10.1186/s12882-021-02361-7 (PMC8066971; doi:10.1186/s12882-021-02361-7)
Supplement: Supplementary file 2 — Additional file 2. [file 12882_2021_2361_MOESM2_ESM.docx]

|  |  | Specialty |  |  |  |  |  |  |  |
| --- | --- | --- | --- | --- | --- | --- | --- | --- | --- |
|  | Nephrology | | | Endocrinology | | | Surgery | | |
|  | conservative | medication | PTx | conservative | medication | PTx | conservative | medication | PTx |
| Age 40, PTH 90, calcium 2.25 | 37.0 | 43.8 | 19.2 | 52.9 | 23.5 | 23.5 | 56.0 | 12.0 | 32.0 |
| Age 65, PTH 90, calcium 2.25 | 38.6 | 47.1 | 14.3 | 68.8 | 18.8 | 12.5 | 56.5 | 17.4 | 26.1 |
| Age 40, PTH 90, calcium 2.8 | 0 | 54.5 | 45.5 | 0 | 25.0 | 75.0 | 21.7 | 26.1 | 52.2 |
| Age 65, PTH 90, calcium 2.8 | 0 | 67.2 | 32.8 | 6.3 | 31.3 | 62.5 | 14.3 | 33.3 | 52.4 |
| Age 40, PTH 40, calcium 2.25 | 92.1 | 7.9 | 0 | 66.7 | 13.3 | 20.0 | 84.2 | 10.5 | 5.3 |
| Age 65, PTH 40, calcium 2.25 | 95.1 | 4.9 | 0 | 80.0 | 13.3 | 6.7 | 84.2 | 10.5 | 5.3 |
| Age 40, PTH 40, calcium 2.8 | 23.3 | 55.0 | 21.7 | 6.7 | 20.0 | 73.3 | 11.1 | 61.1 | 27.8 |
| Age 65, PTH 40, calcium 2.8 | 20.0 | 65.0 | 15.0 | 13.3 | 33.3 | 53.3 | 16.7 | 61.1 | 22.2 |

Supplementary. Frequency in percentages per chosen option for case vignettes, separated by specialty

|  | RHPT related decisions | | |  |  |  |
| --- | --- | --- | --- | --- | --- | --- |
|  | Below median | | | At and above median | | |
|  | conservative | medication | PTx | conservative | medication | PTx |
| Age 40, PTH 90, calcium 2.25 | 49.1 | 24.5 | 26.4 | 39.3 | 42.6 | 18.0 |
| Age 65, PTH 90, calcium 2.25 | 52.0 | 24.0 | 24.0 | 43.1 | 48.3 | 8.6 |
| Age 40, PTH 90, calcium 2.8 | 4.3 | 34.0 | 61.7 | 5.3 | 52.6 | 42.1 |
| Age 65, PTH 90, calcium 2.8 | 4.4 | 40.0 | 55.6 | 3.6 | 67.3 | 29.1 |
| Age 40, PTH 40, calcium 2.25 | 81.4 | 14.0 | 4.7 | 90.6 | 5.7 | 3.8 |
| Age 65, PTH 40, calcium 2.25 | 85.7 | 9.5 | 4.8 | 94.2 | 5.8 | 0 |
| Age 40, PTH 40, calcium 2.8 | 9.8 | 46.3 | 43.9 | 23.5 | 54.9 | 21.6 |
| Age 65, PTH 40, calcium 2.8 | 9.8 | 58.5 | 31.7 | 23.5 | 60.8 | 15.7 |

Supplementary: frequency in percentages per chosen option for case vignettes, dichotomized by number of RHPT related decisions in the past year (median number of RHPT-related decisions was 20)

|  | Affiliation | | |  |  |  |
| --- | --- | --- | --- | --- | --- | --- |
|  | Affiliated hospital | | | Acedemic hospital | | |
|  | conservative | medication | PTx | conservative | medication | PTx |
| Age 40, PTH 90, calcium 2.25 | 35.6 | 37.0 | 27.4 | 57.1 | 28.6 | 14.3 |
| Age 65, PTH 90, calcium 2.25 | 38.2 | 41.2 | 20.6 | 61.0 | 29.3 | 9.8 |
| Age 40, PTH 90, calcium 2.8 | 7.6 | 43.9 | 48.5 | 0 | 43.6 | 56.4 |
| Age 65, PTH 90, calcium 2.8 | 4.8 | 57.1 | 38.1 | 2.6 | 50.0 | 47.4 |
| Age 40, PTH 40, calcium 2.25 | 86.4 | 8.5 | 5.1 | 86.8 | 10.5 | 2.6 |
| Age 65, PTH 40, calcium 2.25 | 89.7 | 6.9 | 3.4 | 91.9 | 8.1 | 0 |
| Age 40, PTH 40, calcium 2.8 | 21.4 | 50.0 | 28.6 | 13.5 | 51.4 | 35.1 |
| Age 65, PTH 40, calcium 2.8 | 21.4 | 58.9 | 19.6 | 13.5 | 59.5 | 27.0 |

Supplementary: frequency in percentages per chosen option for case vignettes, dichotomized by affiliation

|  | Affiliation |  |  |
| --- | --- | --- | --- |
|  | Affiliated hospital | Academic hospital | All respondents |
| PTH concentration (pmol/L) | 110 ± 52 | 101 ± 53 | 107 ± 52 |

Supplementary. PTH concentration (pmol/L ± standard deviation) above which affiliated (n=55) and academic-related (n=35) physicians would opt for a PTx

|  | RHPT-related cases |  |  |
| --- | --- | --- | --- |
|  | Below | At and above | All respondents |
| PTH concentration (pmol/L) | 117 ± 68 | 99 ± 36 | 107 ± 52 |

Supplementary. PTH concentration (pmol/L ± standard deviation) above which the group below (n=38) and the group at and above (n=51) the median amount of RHPT-related cases seen in the last year would opt for a PTx

|  | Years of experience |  |  |
| --- | --- | --- | --- |
|  | Below | At and above | All respondents |
| PTH concentration (pmol/L) | 107 ± 58 | 106 ± 47 | 107 ± 52 |

Supplementary. PTH concentration (pmol/L ± standard deviation) above which the group below (n=41) and the group at and above (n=49) the median amount of years of experience would opt for a PTx
